# Inhibition of plant essential oils and their interaction in binary combinations against tyrosinase

**DOI:** 10.29219/fnr.v66.8466

**Published:** 2022-12-27

**Authors:** Zonglin You, Yonglian Li, Min Chen, Vincent Kam Wai Wong, Kun Zhang, Xi Zheng, Wenfeng Liu

**Affiliations:** 1 School of Biotechnology and Health Sciences, Wuyi University, Jiangmen, China; 2 School of Eco-environment Technology, Guangdong Industry Polytechnic, Guangzhou, China; 3 Dr. Neher’s Biophysics Laboratory for Innovative Drug Discovery, State Key Laboratory of Quality Research in Chinese Medicine, Macau University of Science and Technology, Macau, China

**Keywords:** essential oil, binary combination, interaction, anti-tyrosinase, inhibitory mechanism

## Abstract

**Background:**

Essential oils (EOs), derived from aromatic plants, exhibit properties beneficial to health, such as anti-inflammatory, anti-oxidative, antidiabetic, and antiaging effects. However, the effect of EOs and their interaction in binary combinations against tyrosinase is not yet known.

**Objective:**

To evaluate the underlying mechanisms of EOs and their interaction in binary combinations against tyrosinas.

**Design:**

We explored to investigate the inhibitory effect of 65 EOs and the interaction among cinnamon, bay, and magnolia officinalis in their binary combinations against tyrosinase. In addition, the main constituents of cinnamon, bay, and magnolia officinalis were analyzed by gas chromatography–mass spectrometry (GC–MS).

**Results:**

The results showed that the most potent EOs against tyrosinase were cinnamon, bay, and magnolia officinalis with IC_50_ values of 25.7, 30.8, and 61.9 μg/mL, respectively. Moreover, the inhibitory mechanism and kinetics studies revealed that cinnamon and bay were reversible and competitive-type inhibitors, and magnolia officinalis was a reversible and mixed-type inhibitor. In addition, these results, assessed in mixtures of three binary combinations, indicated that the combination of cinnamon with bay at different dose and at dose ratio had a strong antagonistic effect against tyrosinase. Magnolia officinalis combined with cinnamon or bay experienced both antagonistic and synergistic effect in anti-tyrosinase activity.

**Conclusion:**

It is revealed that natural EOs would be promising to be effective anti-tyrosinase agents, and binary combinations of cinnamon, bay, and magnolia officinalis might not have synergistic effects on tyrosinase under certain condition.

## Popular scientific summary

- 65 plant essential oils have different inhibition against tyrosinas. Cinnamon, bay and magnolia offinalis have most potent inhibition than other EOs. Moreover, cinnamon and bay were reversible and competitive-type inhibitors, and magnolia officinalis was a reversible and mixed-type inhibitor.
- Binary combinations of cinnamon, bay, and magnolia officinalis might not have synergistic effects on tyrosinase under certain condition.

Tyrosinase, a copper-containing enzyme, was reported to play a key role in the synthesis of melanins, which were the pigments responsible for skin color in human beings and enzymatic browning in nature (1). In human being, moderate melanin, catalyzed by tyrosinase, was synthesized to prevent skin lesions caused by ultraviolet radiation. However, the upregulation of tyrosinase activity and excessive accumulation of melanin would cause skin disorders and related diseases, such as freckles, malignant melanoma, and Parkinson’s disease (2–4). In nature, fruits and vegetables browning, related with the increase of tyrosinase activity, was confirmed to shorten the shelf life of fruits and vegetables, which causes an unattractive appearance and unpredicted loss in nutritional quality (5–7). Recent research indicated that kojic acid, widely used for skin whitening and hyperpigmentation preventing, would lead to skin irritation and mutagenic effect on human skin (8–10). Thus, searching for safe tyrosinase inhibitors of nature origin has attracted increasing attention in cosmetic and medicinal industries (11).

Over the years, tyrosinase inhibitors of nature origin were considered free of harmful side effects (7, 12). Therefore, using active constituents derived from natural plants is a promising strategy to improve tyrosinase inhibition activity. Essential oils (EOs), extracted from various aromatic plants, were secondary metabolites (13). They comprised different bioactive components, exhibiting anti-inflammatory, anti-oxidative, antidiabetic, and antiaging properties (14). Recently, the literature on tyrosinase inhibitors from natural source is extensive (12, 15, 16). It was indicated that *Litsea cubeba* EO showed inhibition against tyrosinase with an IC_50_ value of 166.7 μg/mL (17). In addition, EO that extracted from the peel of *C. sinensis oranges* had the low response with 11.18 ± 3.34% inhibition at a concentration of 800 μg/mL (18). However, due to their inadequate potency, most of EOs have not yet used for anti-tyrosinase applications.

Aiming to develop more potent tyrosinase inhibitors from natural source, 65 commercial EOs that extracted from plants were evaluated for their anti-tyrosinase activity as single constituents, as well as in binary combinations in this study. Furthermore, the main components of potent EOs were analyzed by gas chromatography–mass spectrometry (GC–MS). The kinetics and the anti-tyrosinase mechanism of potent EOs were discussed.

## Materials and methods

### Materials

Sixty-five commercial EOs, pure without additive, were purchased from Jingjing Biotechnology Co. (Guangzhou, China). The information of these EOs was listed in Table 1. Tyrosinase (EC 1.14.18.1) and kojic acid were purchased from Sigma-Aldrich (St. Louis, MO). Tyrosinase was dissolved with 0.05 M phosphate buffer solution (PBS, pH 6.81 ± 0.01) and then diluted to 1,500 U/mL. The final concentration of tyrosinase and L-Dopa in PBS was 37.5 U/mL and 1.0 mM, respectively. In addition, kojic acid was used as a standard compound. Other solvents and reagents that had analytical grade were purchased from Tansoole (Shanghai, China).

**Table 1:** The half maximal inhibitory concentration of 65 Eos

No.	Name of EOs	Inhibition at 25 μg/mL (%)	IC_50_ (μg/mL)
1	Ginseng	38.20 ± 2.48	633.2 ± 281.2
2	Burdock	29.96 ± 1.92	425.6 ± 30.5
3	Tangerine peel	37.36 ± 3.97	302.0 ± 5.7
4	Green tea	28.69 ± 2.97	184.8 ± 14.9
5	Bay	53.79 ± 3.19	25.7 ± 5.6
6	Black currant	10.26 ± 1.21	7918.4 ± 1339.6
7	Moringa leaf	25.79 ± 0.22	567.7 ± 86.1
8	Olive	17.01 ± 4.54	2876.9 ± 772.4
9	Papaya	17.47 ± 3.37	3779.7 ± 869.7
10	Tripterygium wilfordii	26.50 ± 2.66	353.1 ± 7.6
11	Moringa seed	26.98 ± 2.83	5299.3 ± 1038.9
12	Celery seed	28.46 ± 6.15	266.8 ± 43.5
13	Ligusticum	30.21 ± 5.92	688.9 ± 32.8
14	Angelica oil	13.22 ± 1.39	1556.4 ± 408.2
15	Frankincense	29.33 ± 1.46	2003.8 ± 315.5
16	Sabal	31.33 ± 3.19	1801.2 ± 349.4
17	Sweet almond	24.19 ± 2.36	708.7 ± 223.5
18	Sacha inchi	17.97 ± 0.99	1260.5 ± 219.3
19	Aloe	22.01 ± 3.68	136.2 ± 15.9
20	Boxthorn seed	34.04 ± 3.50	144.8 ± 10.8
21	Black pepper	12.55 ± 2.16	511.2 ± 32.9
22	Rosewood	31.15 ± 5.08	424.4 ± 30.1
23	Palmarosa	35.77 ± 4.17	1806.3 ± 292.8
24	Cypevol	22.73 ± 1.35	5826.7 ± 489.4
25	Ligusticum Chuan-xiong	4.98 ± 0.06	2807.8 ± 628.0
26	Rosehip	31.57 ± 0.50	796.6 ± 248.3
27	Rose	10.59 ± 4.02	657.2 ± 3.1
28	Agrimonia	37.74 ± 0.48	253.4 ± 46.4
29	Chrysanthemum	31.47 ± 1.06	429.6 ± 95.8
30	Licorice	16.23 ± 4.21	338.7 ± 19.5
31	Carnation	6.80 ± 0.73	671.2 ± 2.2
32	Ginger	37.66 ± 5.97	218.3 ± 35.4
33	Schizandrae fructus	21.17 ± 4.47	747.4 ± 62.6
34	Patchouli	21.48 ± 1.89	290.1 ± 10.9
35	Magnolia officinalis	39.16 ± 4.83	61.9 ± 12.7
36	Ganoderma lucidum spore	11.66 ± 4.22	4375.3 ± 1334.7
37	Fructus Cnidii	13.38 ± 1.74	922.3 ± 179.1
38	Costus	18.42 ± 1.66	2735.5 ± 554.3
39	Seabuckthorn	31.52 ± 0.13	821.1 ± 61.2
40	Seabuckthorn seed	8.67 ± 1.72	3295.3 ± 550.5
41	Peppermint	32.65 ± 5.13	833.4 ± 136.0
42	Zanthoxylum	20.83 ± 4.42	2637.6 ± 739.3
43	Cinnamon	56.03 ± 3.45	30.8 ± 5.6
44	Oleum anisi stellati	23.86 ± 5.28	375.4 ± 44.7
45	Lemongrass	21.76 ± 6.45	534.9 ± 43.2
46	Lavender	37.84 ± 3.84	281.0 ± 48.2
47	Mugwort	5.18 ± 1.87	2378.6 ± 423.5
48	Citronella	28.04 ± 3.88	957.0 ± 292.1
49	Schizonepeta tenuifolia	24.42 ± 2.02	220.4 ± 7.8
50	Lemon	23.75 ± 6.29	547.4 ± 262.2
51	Notopterygium	32.03 ± 6.43	174.7 ± 27.7
52	Honeysuckle	36.39 ± 4.02	223.9 ± 27.8
53	Zedoary turmeric	37.20 ± 3.59	875.1 ± 147.7
54	Pomelo	35.02 ± 1.36	255.5 ± 13.2
55	Bupleurum	32.73 ± 1.03	1357.4 ± 508.3
56	Tulip	28.77 ± 7.04	315.9 ± 54.6
57	Saposhnikovia divaricata	35.97 ± 3.66	224.2 ± 20.7
58	Bergamot	19.45 ± 4.72	902.7 ± 137.9
59	Camellia seed	28.58 ± 4.26	1314.7 ± 71.1
60	Linseed	30.47 ± 0.54	2446.8 ± 547.1
61	Clove	28.21 ± 4.10	458.3 ± 21.7
62	Angelica	37.02 ± 3.03	340.0 ± 24.0
63	Dendrobe	39.24 ± 2.97	389.0 ± 37.6
64	Gallnut	29.75 ± 3.74	211.4 ± 6.7
65	Epimedium	41.63 ± 1.61	189.3 ± 34.5
66	Kojic acid	86.20 ± 0.86	5.30 ± 0.42

### Tyrosinase inhibition assay

All the EOs and kojic acid were dissolved in dimethyl sulfoxide (DMSO) to obtain varying concentrations as required. Inhibitory effect of EOs at different concentrations against tyrosinase was determined as previously described (19). In brief, the mixture containing 85 μL PBS, 10 μL tyrosinase, and 5 μL EOs (DMSO in the blank) was first added to the 96-well plates at 0°C and preincubated for 10 min at 37°C. Second, 100 μL of L-Dopa was rapidly added in the 96-well plates to initiate the enzyme reaction monitored for 60s shake in the microplate reader (Multiskan GO, Thermo Scientific, USA). Finally, the change in absorbance at 475 nm during the 60s shake was tested and recorded. These assays were, respectively, conducted as triplicate, and the concentration required for 50% inhibition of each EO was determined as IC_50_ value. The inhibition rates of EOs were tested at more than 5 concentrations. The IC_50_ values were calculated by the Origin 9.0 software. Inhibition of EOs the tyrosinase reaction was calculated as follows:


$$\text{Inhibition Rate}/\%=\frac{\left(A-B\right)-\left(C-D\right)}{A-B}\times 100\%$$

where A means the absorbance of samples (60s), B means the absorbance of samples (0s), C means the absorbance of the blank (60s), and D means the absorbance of the blank (0s).

### Kinetic analysis of tyrosinase inhibition

As the method reported previously (19), the inhibition mechanism of bay, cinnamon, and magnolia officinalis was determined. Typically, a series of diluted inhibitor solutions were first prepared, at a constant L-Dopa concentration (2 mM). Second, the inhibition of bay, cinnamon, and magnolia officinalis against tyrosinase was measured with different concentrations of tyrosinase (the final: 0.0, 12.5, 25.0, and 37.5 U/mL), respectively.

In addition, the inhibition kinetics of tyrosinase was tested by Lineweaver-Burk plots. A series of diluted inhibitor solutions were obtained, at a constant tyrosinase concentration (37.5 U/mL). The inhibition rates were determined with different concentrations of L-Dopa (the final: 1.5, 1.2, 0.9, 0.6, and 0.3 mM) by using the method reported previously.

### Analysis of 65 EOs with GC-MS

Essential oils were analyzed with GC (TRACE 1300E, Thermo Scientific Corporation, USA) coupled to MS (ISQ Qd, Thermo Scientific Corporation, USA) using a TG-5 MS silica column (30 m × 0.25 mm; film thickness 0.25 μm). The procedure for analyzing GC-MS was shown as follows: helium was first used as the carrier gas, flowing with a rate of 1.0 mL/min. The oven temperature was second programmed at 60°C, with an increase of 5°C/min to 160°C (isotherm at 2 min), and then 10°C/min to 260°C and held for 20 min. The mass spectra were recorded at 70 eV with a scanning range from 35 to 450 m/z. Composition (%) of EOs was calculated in software by using the peak normalization method. Comparing to Kovats retention indices and relative to a C_8_–C_40_ *n*-alkanes standard, the peak identification of different constituents in EOs was determined. Identification of the EOs constituents was performed by comparing the acquired mass spectrum with NIST mass spectral library.

### Statistical analysis

Initially, 65 EOs were tested individually. The three EOs that had the lowest IC_50_ (cinnamon, bay, and magnolia officinalis) were mixed in five different proportions forming each binary combinations (9:1, 3:1, 1:1, 1:3, and 1:9). And then, three binary combinations were tested. In addition, five concentrations (2.8, 8.3, 25.0, 75.0, and 225.0 μg/mL) of three binary combinations were, respectively, determined in order to investigate the mode of interaction among cinnamon, bay, and magnolia officinalis in their binary combinations against tyrosinase. CompuSyn Version 2.0, generating the combination index-fraction affected (CI-Fa) curve, isobologram figure and median effect, was used to classify the anti-tyrosinase activity of cinnamon, bay, and magnolia officinalis in their binary combinations (20). The effects of cinnamon, bay, and magnolia officinalis in their binary combinations were classified according to CI as synergy (0.1 < CI < 0.9), additive (0.9 < CI < 1.1), and antagonism (1.1 < CI < 10) (21).

## Results

### Inhibitory effect of 65 EOs against tyrosinase

It has been reported that several EOs and their main constituents were potential tyrosinase inhibitors (22). However, systematic research of plant EO as natural tyrosinase inhibitors was still lacking. Therefore, we investigated the inhibitory effect of 65 EOs on tyrosinase *in vitro*. As described in Table 1, kojic acid at an initial concentration of 25 μg/mL showed 86.20% inhibition against tyrosinase. Hence, we used kojic acid as the positive control and determined the inhibition of 65 EOs at 25 μg/mL on tyrosinase. The results indicated that most of EOs showed different inhibitory activity against tyrosinase. It was demonstrated that EO of green tea (184.8 ± 14.9 μg/mL) had strongest tyrosinase inhibitory activity in the family *Theaceae*. In the family *Rutaceae*, pomelo (255.5 ± 13.2 μg/mL) showed the strongest inhibition of tyrosinase. The inhibition of plant EOs from the family *Apiaceae* against tyrosinase was in the descending order of notopterygium > saposhnikovia divaricata > agrimonia > celery seed > 300 μg/mL. Aloe from the family *Liliaceae*, ginger from the family *Zingiberaceae*, schizonepeta tenuifolia from the family *Labiatae*, and burdock from the family *Compositae* showed higher inhibition values of 136.2, 218.3, 220.4, and 425.6 μg/mL, respectively. Among these EOs, the most potent EOs against tyrosinase were cinnamon, bay, and magnolia officinalis with IC_50_ values of 25.7, 30.8, and 61.9 μg/mL, respectively (Table 1). Taken together, cinnamon, bay, and magnolia officinalis were selected for further investigation of the anti-tyrosinase mechanism of action.

### Mechanism study

**Fig. 1:**
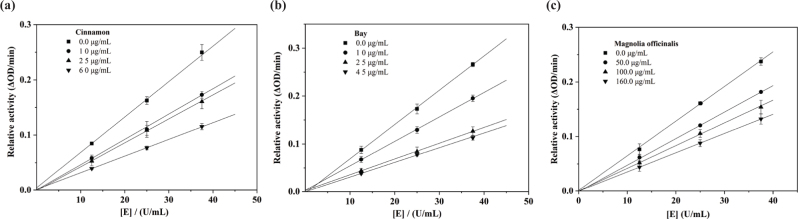
The inhibitory mechanism of cinnamon, bay, and magnolia officinalis against tyrosinase.

To further investigate the inhibitory mechanism of cinnamon, bay, and magnolia officinalis on tyrosinase, the inhibition constants and inhibition types of cinnamon, bay, and magnolia officinalis on tyrosinase activity were determined by using L-Dopa as the substate. As shown in Fig. 1, the four lines were obtained from four different concentrations of cinnamon, bay, and magnolia officinalis. The lines were crossed on the origin. In addition, the slope of the line was gradually decreased with increasing EOs (cinnamon, bay, and magnolia officinalis) concentrations. These results indicated that the inhibition of cinnamon, bay, and magnolia officinalis on tyrosinase activity was reversible.

**Fig. 2:**
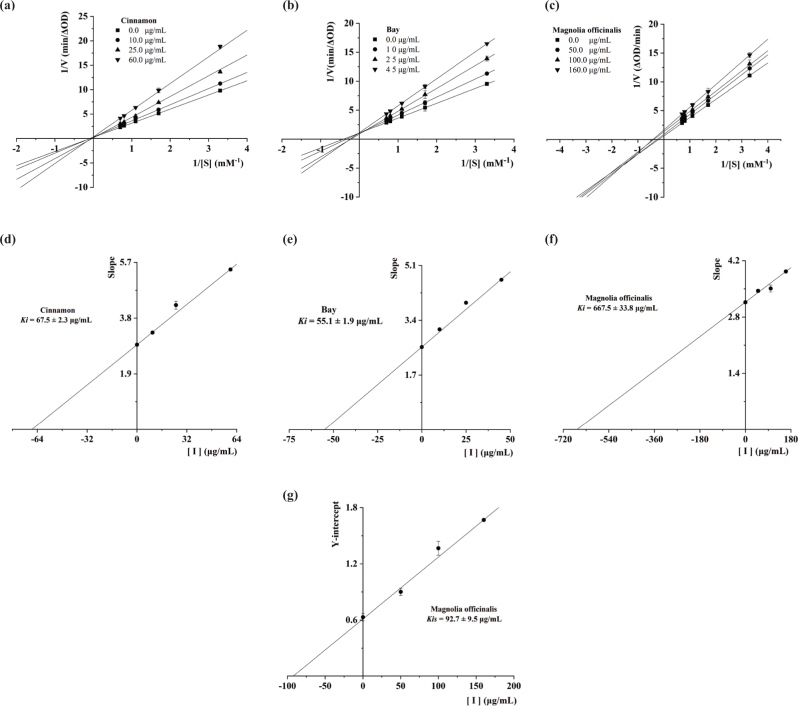
Lineweaver-Burk plots (a, b, and c) for cinnamon, bay, and magnolia officinalis against tyrosinase. (d, e, and f) The plot of slope versus the concentration of cinnamon, bay, and magnolia officinalis for the determination of *K_i_*. (g) The plot of intercept versus the concentration of magnolia officinalis for the determination of *K_is_*.

As seen in Fig. 2, the inhibition kinetics of cinnamon, bay, and magnolia officinalis were analyzed by Lineweaver-Burk plots. The four lines were obtained from four different concentrations of cinnamon and bay (Fig. 2A and B). The lines were crossed on a 1/V axis, indicating that cinnamon and bay exhibited competitive-type inhibition on tyrosinase, respectively. The results of these experiments showed that cinnamon and bay only bound free enzyme and not the enzyme-substrate complex. In addition, the four lines with different slopes were obtained from four different concentrations of magnolia officinalis (Fig. 2C). These lines were crossed on the third quadrant, suggesting that magnolia officinalis showed mixed-type inhibition.

To investigate whether cinnamon, bay, and magnolia officinalis inhibited tyrosinase activity by competitively forming enzyme-inhibitor (EI) complex or interrupting enzyme-substrate-inhibitor (ESI) complex in noncompetitive manner, we determined EI dissociation constants *K_i_* of cinnamon, bay, and magnolia officinalis and the ESI dissociation constants *K_is_* of magnolia officinalis. Inhibition type, and *K_i_* and *K_is_* values of cinnamon, bay, and magnolia officinalis were shown in Table 2. *K_i_* values of cinnamon, bay, and magnolia officinalis were 67.5, 55.1, and 667.5 μg/mL, respectively. Furthermore, a lower value of *K_is_* in comparison with *K_i_* demonstrated that there was weaker binding between enzyme and magnolia officinalis, which suggested preferred noncompetitive over competitive manner. It was confirmed that the cinnamon, bay, and magnolia officinalis had significantly tyrosinase inhibitory activities, indicating that they might be practically used as tyrosinase inhibitors.

**Table 2:** Type of mechanism, and *K_i_* and *K_is_* values of cinnamon, bay, and magnolia officinalis

EOs	Inhibition mechanism	*K_i_* value (μg/mL)	*K_is_* value (μg/mL)
Cinnamon	Competitive type	67.5 ± 2.3	**–**
Bay	Competitive type	55.1 ± 1.9	**–**
Magnolia officinalis	Mixed type	667.5 ± 33.8	92.7 ± 9.5

### Main constituents of cinnamon, bay, and magnolia officinalis

In an attempt to comprehensively characterize the main constituents of the potential EOs that contribute to the anti-tyrosinase activity, three EOs (cinnamon, bay, and magnolia officinalis) were analyzed by GC-MS. As listed in Table 3, the oxygenated fraction (alcohols and aldehydes) accounted for 98.36% of the total cinnamon EO. The main constituent of cinnamon EO was *cis*-cinnamaldehyde (90.85%), followed by 4-phenyl-2-butanol (2.87%), benzyl alcohol (2.41%), diaectone alcohol (2.33%), etc. In bay oil, the oxygenated fraction (alcohols, aldehydes, and acid) accounted for 97.11%, and *trans*-cinnamaldehyde was the most abundant compound (86.03%), followed by *trans*-cinnamic acid (3.55%), diaectone alcohol (3.42%), benzeneacetaldehyde (1.62%), benzaldehyde (1.28%), and α-hexylcinnamalbehyde (1.21%). Finally, magnolia officinalis was characterized by the highest androsta-1,4,6-triene-3,17-dione and magnolol content (32.75 and 31.28% of the total EO, respectively), followed by 4-*O*-methyl honokiol (7.71%), diaectone alcohol (3.72%), 1,4-bis[(1-methylethyl)amino]-9,10-anthracenedione (2.33%), cryptomeridiol (2.10%), etc.

**Table 3:** Chemical components of cinnamon (C), bay (B), and magnolia officinalis (MO)

Chemical components^a^		Content/%				
CAS No.	Name	RI	RI lit.^b^	C	B	MO
14233-37-5	1,4-bis[(1-methylethyl)amino]-9,10-anthracenedione	701	704			2.33
633-35-2	Androsta-1,4,6-triene-3,17-dione	716	722			32.75
528-43-8	Magnolol	900	904			31.48
140-10-3	*trans*-Cinnamic acid	902	902		3.55	
4666-84-6	Cryptomeridiol	910	913			2.10
68592-15-4	4-*O*-Methyl honokiol	916	921			7.71
123-42-2	Diaectone alcohol	920	920	2.23	3.42	3.72
2344-70-9	4-Phenyl-2-butanol	921	928	2.87		
101-86-0	*α*-Hexylcinnamalbehyde	950	951		1.21	
100-52-7	Benzaldehyde	950	952		1.28	
100-51-6	Benzyl alcohol	960	961	2.41		
57194-69-1	*cis*-Cinnamaldehyde	961	962	90.85		
104-55-2	*trans*-Cinnamaldehyde	961	963		86.03	
122-78-1	Benzeneacetaldehyde	962	971		1.62	
	Others			1.64	2.89	19.91

a Major components (content>1%), listed in the order of RI value, are listed in the table.

b Linear retention index is obtained from https://webbook.nist.gov/chemistry/.

### The interaction between cinnamon, bay, and magnolia officinalis in their binary combinations against tyrosinase

The three oils (cinnamon, bay, and magnolia officinalis) with best anti-tyrosinase activity were mixed in five different ratios to perform three binary compositions. The results were shown in Tables 4, 5, and 6. As seen from data in Table 4, the combination cinnamon + bay had a high combination index (CI >1.1), indicating that cinnamon combined bay at different dose (2.8, 8.3, 25, 75, and 225 μg/mL) and dose ratios (9:1, 3:1, 1:1, 1:3, and 1:9) had an antagonistic effect on tyrosinase. Furthermore, the result in Table 4 revealed that low dose of cinnamon + magnolia officinalis (2.8 and 8.3 μg/mL) showed an antagonistic effect on tyrosinase. However, moderate dose (25 μg/mL) and high dose (75 and 225 μg/mL) of cinnamon + magnolia officinalis experienced antagonistic, additive, and synergistic effects on tyrosinase. In addition, CI values of bay + magnolia officinalis at low dose (2.8 and 8.3 μg/mL) and different dose ratios against tyrosinase were ranged from 1.0 to 1.2, suggesting that the combination of bay + magnolia officinalis had antagonistic and additive effect on tyrosinase, respectively. Combination of bay and magnolia officinalis at 25 and 225 μg/mL showed additive and synergistic effect on tyrosinase. Bay combined with magnolia officinalis at 75 μg/mL and different dose ratios had a synergistic effect against tyrosinase.

**Table 4:** The interaction between cinnamon and bay against tyrosinase

Dose (μg/mL)	Cinnamon:bay									
	9:1		3:1		1:1		1:3		1:9	
	CI	Effect^a^	CI	Effect^a^	CI	Effect^a^	CI	Effect^a^	CI	Effect^a^
2.8	2.95	Ant.	2.63	Ant.	2.42	Ant.	2.17	Ant.	1.71	Ant.
8.3	2.37	Ant.	2.06	Ant.	1.85	Ant.	1.68	Ant.	1.54	Ant.
25	1.81	Ant.	1.91	Ant.	1.56	Ant.	1.38	Ant.	1.28	Ant.
75	1.99	Ant.	1.83	Ant.	1.59	Ant.	1.56	Ant.	1.46	Ant.
225	2.50	Ant.	1.92	Ant.	2.02	Ant.	1.81	Ant.	1.72	Ant.

a Ant. means antagonism.

**Table 5:** The interaction between cinnamon and magnolia officinalis against tyrosinase

Dose (μg/mL)	Cinnamon:magnolia officinalis									
	9:1		3:1		1:1		1:3		1:9	
	CI	Effect^a^	CI	Effect^a^	CI	Effect^a^	CI	Effect^a^	CI	Effect^a^
2.8	7.30	Ant.	6.20	Ant.	4.38	Ant.	3.29	Ant.	2.61	Ant.
8.3	3.16	Ant.	3.93	Ant.	3.70	Ant.	4.16	Ant.	4.06	Ant.
25	0.81	Syn.	1.53	Ant.	2.07	Ant.	1.95	Ant.	1.89	Ant.
75	0.56	Syn.	0.68	Syn.	1.08	Addit.	1.55	Ant.	1.94	Ant.
225	0.24	Syn.	0.43	Syn.	0.59	Syn.	0.70	Syn.	1.04	Addit.

a Ant. means antagonism, Addit. means additive, and Syn. means synergy.

**Table 6:** The interaction between bay and magnolia officinalis against tyrosinase

Dose (μg/mL)	Bay:magnolia officinalis									
	9:1		3:1		1:1		1:3		1:9	
	CI	Effect^a^	CI	Effect^a^	CI	Effect^a^	CI	Effect^a^	CI	Effect^a^
2.8	1.28	Ant.	1.20	Ant.	1.18	Ant.	1.18	Ant.	1.00	Addit.
8.3	1.07	Addit.	1.55	Ant.	1.51	Ant.	1.72	Ant.	1.78	Ant.
25	0.74	Syn.	1.04	Addit.	1.01	Addit.	0.92	Syn.	0.90	Addit.
75	0.53	Syn.	0.89	Syn.	0.79	Syn.	0.68	Syn.	0.77	Syn.
225	0.44	Syn.	0.87	Syn.	0.90	Syn.	0.78	Syn.	0.90	Addit.

a Ant. means antagonism, Addit. means additive, and Syn. means synergy.

## Discussion

Tyrosinase, known as monophenol and polyphenol oxidase, is responsible for synthesis of melanins in plants and animals (23). In the process of melanin biosynthesis, tyrosinase catalyzed the oxidation of mono- or di-phenolic compounds to corresponding L-Dopa and dopaquinones (24). Previous studies confirmed that several compounds, including arbutin, azelaic acid, and kojic acid, had been widely used as hyperpigmentation preventing agents and whitening agents (25). Nevertheless, kojic acid, widely used in the cosmetic products, had profound drawbacks, such as skin irritation and mutagenic effect on mammalian cells (26). Therefore, more attention is urgently needed to searching for safe tyrosinase inhibitors. In the present study, we investigated whether the EOs derived from natural plants as single constituent as well as in binary combinations had inhibitory effect on tyrosinase. Moreover, the kinetics and the anti-tyrosinase mechanism of potent EOs (cinnamon, bay, and magnolia officinalis) were determined by using L-Dopa as the substrate. Additionally, the main components of cinnamon, bay, and magnolia officinalis were analyzed by GC-MS.

It was indicated that EOs and their main constituents had a wild range of bioactivities, such as antibacterial, antifungal, and anti-inflammatory activities (5, 13). Herein, we investigated inhibitory effect of 65 EOs on tyrosinase *in vitro*. The results demonstrated that most of EOs showed different inhibitory activity against tyrosinase. Furthermore, pomelo, notopterygium, aloe, ginger, schizonepeta tenuifolia, and burdock showed higher inhibition than that of other EO from the families of *Rutaceae*, *Apiaceae*, *Liliaceae*, *Zingiberaceae*, *Labiatae*, and *Compositae*, respectively. Among these EOs, the most potent EOs against tyrosinase were cinnamon, bay, and magnolia officinalis with IC_50_ values of 25.7, 30.8, and 61.9 μg/mL, respectively. Furthermore, GC-MS analysis showed that *cis*-cinnamaldehyde (90.85%), *trans*-cinnamaldehyde (86.03%), androsta-1,4,6-triene-3,17-dione (32.75%), and magnolol (31.28%) were the main constituents of cinnamon, bay, and magnolia officinalis, respectively. Previous studies confirmed that these compounds had anti-tyrosinase activity (27, 28). Thus, cinnamon, bay, and magnolia officinalis might be responsible for anti-tyrosinase activity of these compounds.

As shown in Fig. 1, the inhibition of cinnamon, bay, and magnolia officinalis on tyrosinase activity was reversible. Moreover, further studies demonstrated that cinnamon and bay exhibited competitive-type inhibition on tyrosinase, and magnolia officinalis showed mixed-type inhibition. Cinnamon and bay only bound free enzyme and not the enzyme-substrate complex. The inhibitory mechanism revealed that *K_i_* values of cinnamon, bay, and magnolia officinalis were 67.5, 55.1, and 667.5 μg/mL, respectively. It was confirmed that there was weaker binding between enzyme and magnolia officinalis, which suggested preferred noncompetitive over competitive manner. Taken together, these results demonstrated that the cinnamon, bay, and magnolia officinalis showed effectively anti-tyrosinase activity, indicating that they could be used as natural tyrosinase inhibitors.

Our studies revealed that the combination cinnamon + bay had a high (CI >1.1), indicating that cinnamon combined bay at different dose (2.8, 8.3, 25, 75, and 225 μg/mL) and dose ratios (9:1, 3:1, 1:1, 1:3, and 1:9) had an antagonistic effect on tyrosinase. Low dose of cinnamon + magnolia officinalis (2.8 and 8.3 μg/mL) at five dose ratios showed an antagonistic effect on tyrosinase. In addition, bay combined with magnolia officinalis at 75 and 225 μg/mL and different dose ratios had synergistic and additive effects on tyrosinase. The possible reason for these was *cis*- and *trans*-cinnamaldehyde, and principal compounds of cinnamon and bay EO, mixed with different dose and different dose ratios exerted an antagonistic effect on tyrosinase. Combination of *cis*-cinnamaldehyde from cinnamon and major constituents of magnolia officinalis (androsta-1,4,6-triene-3,17-dione and magnolol) at 2.8 and 8.3 μg/mL exhibited an antagonistic effect on tyrosinase, respectively. Moreover, combination of *trans*-cinnamaldehyde from cinnamon with major constituents of magnolia officinalis at 75 and 225 μg/mL exhibited an antagonistic effect on tyrosinase, which showed synergistic and additive effect on tyrosinase. Herein, investigating the anti-tyrosinase effect of the combination of main constituents in bay, cinnamon, and magnolia officinalis EO may provide theoretical basis for searching novel natural tyrosinase inhibitors as whitening agents in cosmetics products. However, the interactions of combination of these compounds were currently investigated in the ongoing studies in our lab.

In summary, we indicated that natural tyrosinase inhibitors could be discovered from EOs of naturally edible plants. Among the EOs, cinnamon, bay, and magnolia officinalis significantly inhibited tyrosinase activity. The *in vitro* experiments demonstrated that cinnamon and bay were reversible and competitive-type inhibitors, and magnolia officinalis was a reversible and mixed-type inhibitor. Additionally, the further studies revealed that binary combinations of cinnamon, bay, and magnolia officinalis might not have synergistic effects on tyrosinase under certain condition. Collectively, these results demonstrated that cinnamon, bay, and magnolia officinalis EOs, promising tyrosinase inhibitors, could be used as whitening agents in cosmetics products.
